# Transferable *optrA-*mediated linezolid resistance in enterococci from high-altitude river sediments of Gilgit-Baltistan, Pakistan

**DOI:** 10.1128/aem.01129-26

**Published:** 2026-09-02

**Authors:** Serena Simoni, Andrea Brenciani, Alessandra Di Gregorio, Francesca Romana Massacci, Lucilla Cucco, Maisoor Ahmed Nafees, Saif Ud Din, Nelofar Hanif, Stefania Gorbi, Francesco Regoli, Eleonora Giovanetti, Carla Vignaroli

**Affiliations:** 1 Department of Life and Environmental Sciences, Polytechnic University of Marche 9294 , Ancona, Italy; 2 Department of Biomedical Sciences and Public Health, Polytechnic University of Marche 9294 , Ancona, Italy; 3 Department of Research and Development, Istituto Zooprofilattico Sperimentale dell’Umbria e delle Marche ‘Togo Rosati’ 34399, Perugia, Italy; 4 Department of Animal Sciences, Karakoram International University 72596, Gilgit, Pakistan; Norwegian University of Life Sciences, Ås, Norway

**Keywords:** oxazolidinone resistance genes, *optrA*, plasmids, conjugation, enterococci

## Abstract

Environmental dissemination of antimicrobial resistance is a growing global concern. This study investigated the occurrence, genetic context, and mobility of the oxazolidinone resistance genes in enterococci isolated from high-altitude river sediments in the Gilgit-Baltistan region of Pakistan. A total of 17 florfenicol-resistant enterococci were recovered from 3 of the 12 sampling sites analyzed. Four multidrug-resistant strains (two *Enterococcus faecium*, one *E. faecalis*, and one *E. hirae*) harboring the *optrA* gene were selected for whole-genome sequencing using Illumina and Nanopore platforms. Genomic analyses showed that *optrA* was plasmid-borne in all isolates and co-localized with additional resistance genes, including macrolide, phenicol, and tetracycline resistance determinants. The *optrA* genetic context was frequently associated with insertion sequences, suggesting their role in the mobilization of multidrug-resistance elements. Mini-translocatable units of the *optrA* genetic context were detected as circular forms in two of four strains, further supporting their mobility potential. Conjugation assays demonstrated successful horizontal transfer of the *optrA* gene mediating linezolid resistance, with transfer frequencies ranging from 2.03 × 10⁻⁶ to 6.36 × 10⁻⁴ transconjugants per recipient cell. *optrA*-carrying plasmids remained stable after serial passages without antibiotic pressure. Phylogenetic analysis showed that isolates belonged to distinct sequence types and clustered with strains of diverse geographic and ecological origins, suggesting dissemination of *optrA* across different genetic backgrounds rather than clonal expansion. The occurrence of linezolid-resistant enterococci in high-altitude river sediments suggests their widespread distribution and emphasizes the need for broader environmental surveillance programs to monitor clinically relevant resistances even in remote ecosystems.

**IMPORTANCE:**

This study provides important insights into the environmental dissemination of antimicrobial resistance by demonstrating the occurrence and genetic diversity of clinically relevant resistance determinants in enterococci isolated from remote high-altitude river sediments. The findings emphasize that natural environments could serve as reservoirs and exchange hubs for antimicrobial resistance, even in ecosystems with limited direct anthropogenic impact. Therefore, integrated One Health surveillance strategies that include environmental compartments alongside human and animal populations are required to better monitor and mitigate the emergence of clinically significant resistant bacteria.

## INTRODUCTION

In recent years, the occurrence of antibiotics in the environment and the associated development of antimicrobial resistance in microorganisms have emerged as major issues of scientific and public concern (1). The increasing prevalence of antimicrobial resistance is widely recognized to be closely linked to the inappropriate use and widespread overuse of antibiotics in human medicine, livestock production, and agriculture (2).

Multiple sources contribute significantly to the environmental burden of antibiotics, including hospital and domestic waste, wastewater treatment plants, improper disposal of unused pharmaceuticals, veterinary applications, and agricultural practices such as livestock production and aquaculture (3, 4).

Antibiotic pollution imposes selective pressure on bacterial populations, thereby promoting the emergence and dissemination of antibiotic resistance genes through horizontal gene transfer (HGT) (5). Evidence of horizontal transfer of resistance determinants between environmental bacteria and human pathogens underscores the critical role of the environmental resistome (6).

Enterococci are common members of the intestinal microbiota of humans and animals and are released into the environment in large numbers through fecal shedding. As a result, they can be detected in a wide range of environmental niches, including soil, animal-derived foods, plants, and aquatic environments (7).

Enterococci have long been used as a fecal indicator organism for routine water quality monitoring, and this approach has subsequently been extended to food matrices (8). In recent years, enterococci have also gained attention as potential indicators for the surveillance of antibiotic resistance within the One Health perspective (9).

Although *Enterococcus* spp. are generally considered commensal organisms, they represent major causes of nosocomial infections worldwide (10). The increasing prevalence of acquired antimicrobial resistance severely limits therapeutic options, and oxazolidinones remain among the few last-resort antibiotics recommended for the treatment of serious infections caused by vancomycin-resistant enterococci (VRE) and multidrug-resistant (MDR) enterococci (11).

Oxazolidinones (linezolid and tedizolid) inhibit bacterial protein synthesis by binding to the 50S ribosomal subunit (12, 13). However, several mechanisms conferring resistance or reduced susceptibility to oxazolidinones have been identified, including ribosomal mutations (23S rRNA and/or L3 and L4 ribosomal proteins) and the acquisition of transferable resistance genes, such as *cfr*/*cfr*-like, *optrA*, *poxtA*, and *poxtA2* (12, 13).

Remarkably, in enterococci, linezolid resistance genes are often carried by mobile genetic elements and are readily transferred between bacteria via HGT (12, 13).

The *optrA* gene was first identified in the linezolid-resistant *Enterococcus faecalis* strain E349 of clinical origin in China (14) and is currently recognized as the predominant acquired oxazolidinone resistance determinant in enterococci, particularly in *E. faecalis* (15). *optrA* encodes an ABC-F protein that determines resistance to oxazolidinones and phenicols through a ribosomal protection mechanism (16).

Although the *optrA* gene was initially identified in a human *Enterococcus* isolate, it has since been reported worldwide in bacteria from diverse sources, including animals, foods of animal origin, urban wastewaters, plant-derived products, and natural environments, such as marine and freshwater ecosystems, highlighting its broad ecological distribution (17–25).

Recent studies conducted in the Gilgit-Baltistan region have revealed a significant lack of awareness among the general population regarding the appropriate use of antibiotics and the risks of antimicrobial resistance, largely linked to self-medication practices. These factors make the region particularly susceptible to the emergence and dissemination of antimicrobial resistance (26). In this context, investigating specific antimicrobial resistance determinants, including oxazolidinone resistance genes, becomes especially relevant. However, studies investigating the occurrence of oxazolidinone resistance genes in Pakistan remain scarce. To date, only a few studies have examined the presence of linezolid resistance genes in Pakistan, underscoring the limited understanding of their environmental distribution in this country (27).

Therefore, the aim of this study was to investigate the presence of linezolid resistance genes in enterococci isolated from river sediment samples collected at different sites in the Gilgit-Baltistan region of Pakistan.

## RESULTS

### Detection of oxazolidinone resistance genes in florfenicol-resistant enterococci and antimicrobial susceptibility profiles

**Fig 1:**
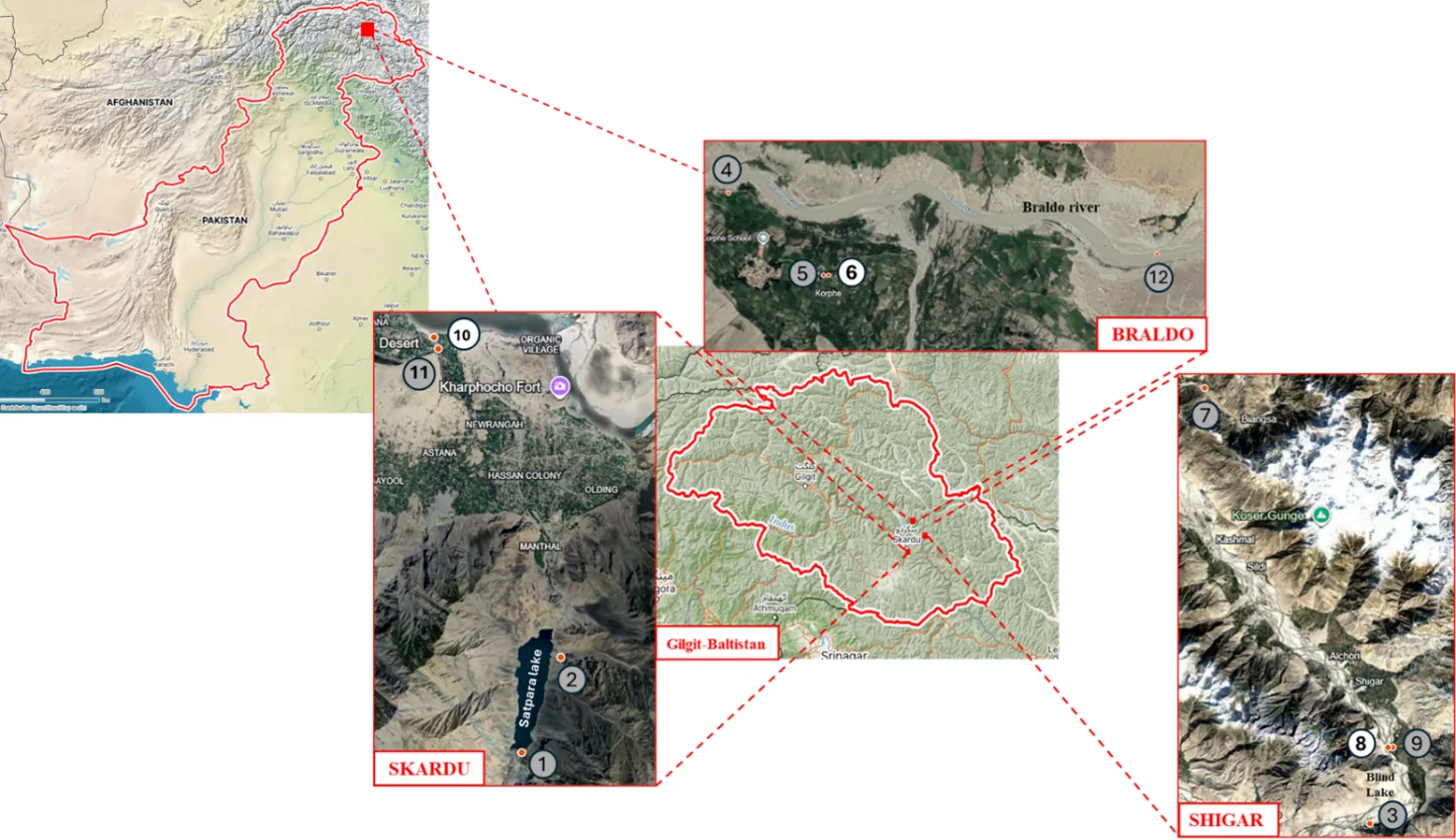
River sediment sampling sites from (1) to (12), across the three macroareas of the Gilgit-Baltistan region, Pakistan. White circles indicate locations where florfenicol-resistant enterococci were recovered. Geographic coordinates and altitude (m a.s.l.) of each sampling site were as follows: (1) 35°12′42″ N 75°37′34″ E, 2637; (2) 35°14′12″ N 75°38′23″ E, 2672; (3) 35°21′48″ N 75°41′48″ E, 2181; (4) 35°40′42″ N 75°48′32″ E, 2898; (5) 35°40′30″ N 75°48′51″ E, 2997; (6) 35°40′30″ N 75°48′51″ E, 2997; (7) 35°42′38″ N 75°32′23″ E, 2398; (8) 35°25′27″ N 75°43′06″ E, 2249; (9) 35°25′27″ N 75°42′50″ E, 2243; (10) 35°19′17″ N 75°35′54″ E, 2160; (11) 35°19′06″ N 75°35′59″ E, 2171; and (12) 35°40′33″ N 75°49′50″ E, 2999. Base imagery from Google Earth; Google/Landsat, Copernicus, SIO, NOAA, U.S. Navy, NGA, and GEBCO (imagery date: 1 January 2021). Modified by the authors.

From river sediments collected at sampling sites 6, 8, and 10 (Fig. 1), 17 florfenicol-resistant enterococci were selected on Slanetz-Bartley agar plates supplemented with florfenicol. All isolates tested positive for the *optrA* gene and were analyzed by *Sma*I pulsed-field gel electrophoresis (PFGE) to compare their genetic profile and identify closely related and clonal isolates. PFGE analysis revealed only four distinct pulsotypes, and therefore, one representative isolate from each pulsotype was identified (*Enterococcus faecium* 10M, *E. faecium* 8J, *E. faecalis* 6AS, and *Enterococcus hirae* 10I) and selected for further characterization.

The four enterococci were resistant to florfenicol (MIC, all 128 mg/L), linezolid (MIC range, 8–16 mg/L), erythromycin (MIC, all >128 mg/L), chloramphenicol (MIC range, 32–64 mg/L), and tetracycline (MIC range, 64–128 mg/L), while they were susceptible to vancomycin (MIC range, 0.25–2 mg/L). *E. faecalis* 6AS showed high-level gentamicin resistance (MIC, >512 mg/L) (Table 1).

**TABLE 1:** Antibiotic susceptibility of the enterococcal isolates characterized in this study^*a*^

Strain	Isolation data			MIC (mg/L)						
	Year	Source	Sampling zone	LZD	VAN	TET	ERY	FFC	CHL	GEN
*E. faecalis* 6AS	2024	Sediment river	Askole stream	8	1	64	>128	128	64	>512
*E. faecium* 8J	2024	Sediment river	Shigar minor stream	16	2	128	>128	128	64	8
*E. faecium* 10M	2024	Sediment river	Hargisa stream	8	0.25	128	>128	128	64	16
*E. faecium* 10I	2024	Sediment river	Hargisa stream	8	0.25	128	>128	128	32	8

^*a*^ LZD, linezolid; VAN, vancomycin; TET, tetracycline; ERY, erythromycin; FFC, florfenicol; CHL, chloramphenicol; GEN, gentamicin.

### Genomic analysis

To analyze the resistome, virulome, plasmidome, sequence type (ST), phylogenesis, and the *optrA* genetic context, the four enterococci were subjected to whole-genome sequencing (WGS) analysis. The main genetic characteristics are reported in Table 2.

**TABLE 2:** Genetic characteristics of the four enterococci investigated in this study

Isolate	Sequence type	Plasmids (p) and chromosome (chr)	Size (kb)	rep gene	Plasmid family or group	T4SS conjugative type^*a*^	Relaxase (MOB), ATPase (virb, traU), and coupling protein (t4cp)^*b*^	Acquired resistance genes				
								Aminoglycosides	Macrolides, lincosamides, streptogramins group B	Tetracyclines	Diaminopyrimidines	Amphenicols, oxazolidinones
*E. faecalis* 6AS	ST2175 (novel)	**pEfa6AS-optrA**	67.6	rep9b	RepA_N	–^*c*^	MOBP1, t4cp2	–	*erm*(B)	–	–	*fexA, optrA*
		p6AS-3	66.9	repUS11	Inc18	T4SS_typeFATA	MOBP1, virb4, t4cp2	–	*erm*(B)	–	–	–
		p6AS-4	53.6	rep1/repUS1	Inc18	dCONJ_typeFATA	MOBB, virb4	*aac(6′)-aph(2″), ant(6)-Ia*	*erm*(B)	–	–	–
		chr	2,780	–	–	–	–	–	*lsa*(A)	*tet*(M)	–	–
*E. faecium* 8J	ST3063 (novel)	**pDY28-optrA-like**	56.2	–	–	T4SS_typeFATA	MOBB, virb4, t4cp1	–	–	–	–	*fexA, optrA*
		p8J-3	272.1	repUS15/rep22	Multi-replicon	T4SS_typeFA	MOBB, virb4, t4cpA	*ant(6)-Ia*	*lsa*(E), *lnu*(B), *erm*(B)	*tet*(M), *tet*(L)	–	–
		p8J-8	5.7	rep18b	Rep3	–	–	–	–	–	–	–
		chr	2,687	–	–	–	–	*aac(6')-Ii*	*msr*(C)	–	*dfrK*	–
*E. faecium* 10M	ST12	**pEfm10M-optrA**	32.6	repUS12/rep2	Multi-replicon	–	MOBB	–	*erm*(B)	*tet*(M), *tet*(L)	–	*fexA, optrA*
		p10M-24	213	repUS15	RepA_N	T4SS_typeT	MOBB, virb4, t4cp1	*ant(6)-Ia*	*lsa*(E), *lnu*(B)	–	–	–
		chr	2,459	–	–	–	–	*aac(6')-Ii*	*msr*(C)	–	–	–
*E. hirae* 10I		**pEhi10I-optrA**	27.3	repUS1	Inc18	–	–	–	*erm*(A)-like	–	–	*fexA, optrA*
		p10I-2	36.6	rep2/repUS12	Multi-replicon	–	MOBB	–	*erm*(B)	*tet*(M), *tet*(L)	–	–
		chr	2,685	–	–	–	–	*aac(6′)-Iid*	*lnu*(G)	–	–	–

^*a*^ Conjugative and decayed conjugative (dCONJ) type 4 secretion system (T4SS) detected by CONJscan tool (https://galaxy.pasteur.fr/).

^*b*^ Mandatory proteins common to all conjugative system types detected by CONJscan tool: MOB (relaxase protein), t4cp (coupling protein), and virb4 or traU (ATPase).

^*c*^ –, corresponding genetic characteristic was not detected.

MLST revealed that the *E. faecalis* 6AS and *E. faecium* 8J belonged to the novel sequence types ST2175 and ST3036, respectively, whereas *E. faecium* 10M was assigned to ST12. The two *E. faecium* strains were classified within clade A2 based on average nucleotide identity (ANI > 99%) with A2 reference genomes (28). Clade A2 typically includes environmental, animal, and human-associated strains and is distinct from clade A1, which comprises hospital-associated epidemic strains (28).

Overall, PlasmidFinder analyses revealed that each strain harbored two to three plasmids. Moreover, the CONJscan tool (https://galaxy.pasteur.fr/) was used to screen all plasmids for genes encoding key components of conjugation systems and to classify them as conjugative or mobilizable (Table 2).

ResFinder analyses showed that each strain carried numerous acquired antibiotic resistance genes in addition to linezolid resistance determinants, most of which were located on different plasmids (Table 2).

Regarding linezolid resistance, no mutations affecting the 23S rRNA or the L3/L4 ribosomal proteins were detected either in the assembled genomes or via the LRE-finder analysis.

All strains, except for *E. hirae*, carried chromosomally encoded virulence genes (*efa* and *ace*) characteristic of *E. faecium* and *E. faecalis* species. *E. faecalis* 6AS additionally harbored several virulence factors, including those involved in biofilm formation (*ebp*ABC operon), gelatinase production (*gelE*), hyaluronidase activity (*hylA* and *hylB*), aggregation substance (*prgB*), sex pheromone production (cCF10, cOB1, cAD, and cAME), and oxidative stress response (*tpx*).

### Analysis of the *optrA*-carrying genetic elements

Bioinformatic analysis showed that both *E. faecalis* 6AS and *E. faecium* 10M harbored an OptrA protein identical to the wild-type OptrA_E394_ first found in a human clinical *E. faecalis* (accession no. KP399637) isolated in China (14). In contrast, *E. faecium* 8J and *E. hirae* 10I displayed the OptrA variants EYKWDVKELYNKQLEIG and KLD, respectively, which have also been previously reported in enterococcal populations (12, 29) (Table 3).

**TABLE 3:** Analysis of the *optrA* carrying plasmids in the four enterococci investigated in this study

Strain	Antibiotic resistance genes	OptrA protein variant	*optrA* genetic context	Genetic element (GenBank accession no.)	Rep family plasmid	Size (bp)	Reference
*E. faecalis* 6AS	***optrA***, *fexA*, *erm*(B)	WT^*a*^	ΔTn*6373*	pEfa6AS-*optrA* (PZ148336)	repA_N	67,629	This study
*E. faecium* 8J	***optrA***, *fexA*	EYKWDVKELYNKQLEIG^*b*^	ΔTn*558*	pDY28-*optrA*-like	–^*e*^	56,270	(30)
*E. faecium* 10M	***optrA***, *fexA*, *erm*(B), *tet*(M)/(L)	WT	*optrA/fexA* PCTn^*c*^	pEfm10M-*optrA* (PZ148334)	Multi-replicon	32,640	This study
*E. hirae* 10I	***optrA***, *fexA*, *erm*(A)-like	KLD^*d*^	*optrA/fexA/erm*(A)-like PCTn	pEhi10I-*optrA* (PZ148333)	Inc18	27,256	This study

^*a*^ WT, wild type.

^*b*^ Positions of OptrA protein mutations in *E. faecium* 8J (the mutated amino acid is indicated in bold): K3**E**, N12**Y**, N122**K**, Y135**W**, Y176**D**, A350**V**, Q509**K**, Q541**E**, M552**L**, N560**Y**, K562**N**, Q565**K**, E614**Q**, I627**L**, D633**E**, N640**I**, and R650**G**.

^*c*^ PCTn, pseudo-compound transposon flanked by IS1216 elements.

^*d*^ Positions of OptrA protein mutations in *E. hirae* 10I (the mutated amino acid is indicated in bold): T112**K**, S147**L**, and Y176**D**.

^*e*^ –, plasmid could not be assigned to a known Rep family.

In all four enterococcal isolates, the *optrA* gene was plasmid located, although embedded within different genetic environments and plasmid backbones (Table 3).

**Fig 2:**
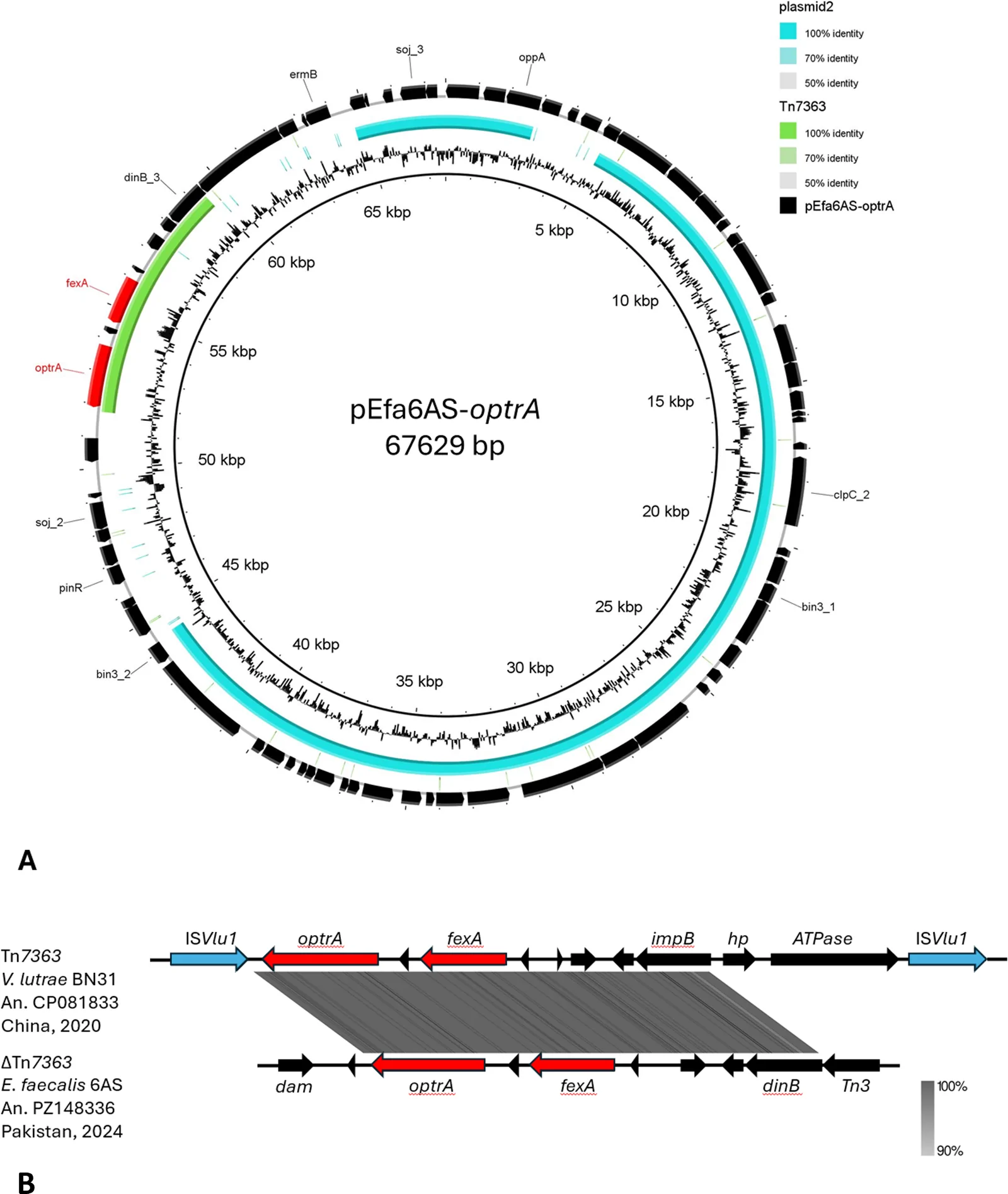
Schematic representation of the pEfa6AS-*optrA* plasmid and the *optrA* genetic context in *E. faecalis* 6AS (PZ148336). (**A**) Plasmid was aligned with the similar plasmid 2 (LR962798.1) previously identified in *E. faecalis* 28157_4#153 and the Tn*7363* transposon (CP081833) using BRIG software. Arrows indicate the positions and directions of transcription of the different genes; some antibiotic resistance determinants and relevant genes described in this study are shown in red. (**B**) Linear maps of the *optrA*-carrying ΔTn7363 of *E. faecalis* 6AS (PZ148336) with the Tn*7363* transposon (CP081833) of the *Vagococcus lutrae* BN31 strain. The figure was created using the Easyfig tool (https://mjsull.github.io/Easyfig/). The positions and transcriptional direction of the ORFs are represented with arrows.

In *E. faecalis* 6AS, *optrA* was co-localized with *erm*(B) and *fexA* on a novel 67,629 bp repA_N type plasmid, designated pEfa6AS-*optrA* (accession no. PZ148336). This plasmid showed high nucleotide similarity (99.51% identity; 67% coverage) with the uncharacterized plasmid 2 (accession no. LR962798.1) previously identified in *E. faecalis* 28157_4#153 strain from the Netherlands (Fig. 2A). No T4SS, associated with conjugation, was detected, although genes encoding a coupling protein and a relaxase were present, indicating that the plasmid was likely a mobilizable element (Table 2). The major ORFs' features are detailed in Table S1. The *optrA* gene was co-localized with *fexA* within a 7,605 bp region identical (100% nucleotide identity; 53% coverage) to a portion of the pseudo-compound transposon Tn*7363* (14,312 bp; accession no. CP081833), first described in *Vagococcus lutrae* from a pig lung sample in China (Fig. 2B) (31). However, the ΔTn*7363* of *E. faecalis* 6AS lacked two ORFs encoding a hypothetical protein and an ATPase, as well as the two IS*Vlu1* transposases that normally flank Tn*7363* in *V. lutrae*.

**Fig 3:**
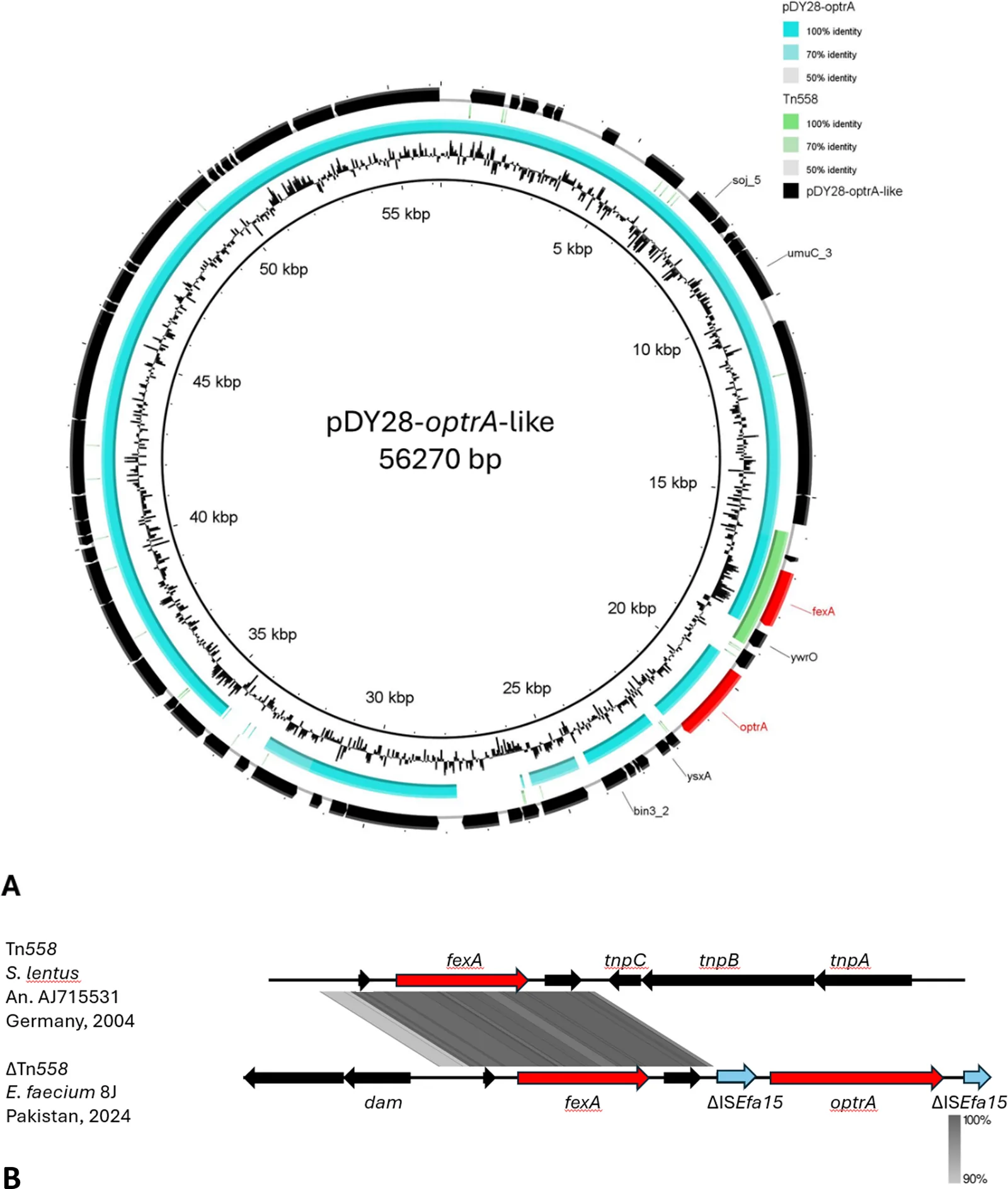
Schematic representation of the pDY28-*optrA*-like plasmid and the *optrA* genetic context in *E. faecium* 8J. (**A**) Plasmid was aligned with the similar reported plasmid pDY28-*optrA* (MW207670.1) and Tn*558* transposon (AJ715531) using BRIG software. Arrows indicate the positions and directions of transcription of the different genes; some antibiotic resistance determinants and relevant genes described in this study are shown in red. (**B**) Linear maps of the *optrA*-carrying *ΔTn558* of *E. faecium* 8J with the Tn*558* transposon (AJ715531) of the *Staphylococcus lentus* 8 strain. The figure was created using the Easyfig tool (https://mjsull.github.io/Easyfig/). The positions and transcriptional direction of the ORFs are represented with arrows.

In *E. faecium* 8J, *optrA* was located on a 56,270-bp plasmid that did not belong to any known replicon family and showed high similarity (97.10% nucleotide identity; 87% coverage) to plasmid pDY28-*optrA* (54,975 bp; accession no. MW207670.1), previously detected in the swine-derived *E. faecium* strain (30) (Fig. 3A). The plasmid-contained genes matched with a conjugative T4SS_type of the FATA family (according to CONJscan classification) (Table 2), and *optrA* was co-localized with *fexA* within a truncated Tn*558* transposon, originally described in *Staphylococcus lentus* (accession no. AJ715531) (32). In the ΔTn*558*, the *optrA* gene was flanked by two truncated IS*Efa15* elements and lacked the *tnpA*, *tnpB*, and *tnpC* transposases of the prototype Tn*558* (Fig. 3B).

**Fig 4:**
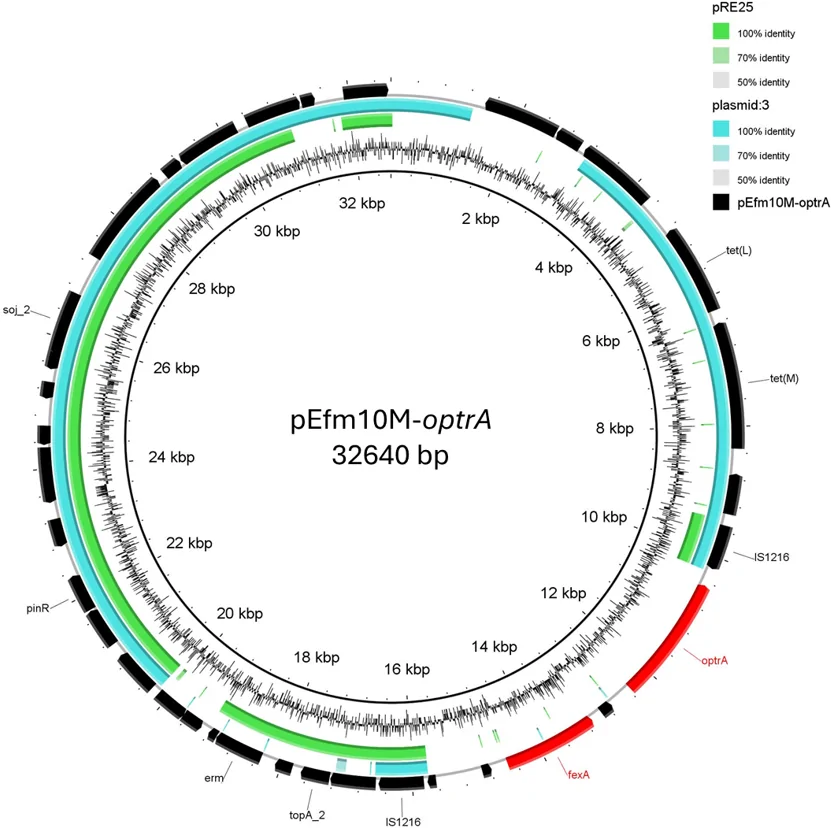
Schematic representation of pEfm10M-*optrA* plasmid (PZ148334) in *E. faecium* 10M. Plasmid was aligned with the similar plasmid 3 (LR134097.1) previously identified in *E. faecium* E1334 and the *E. faecalis* pRE25 (X92945) using BRIG software. Black arrows indicate the positions and orientations of genes; some antibiotic resistance determinants and relevant genes described in this study are shown in red.

In *E. faecium* 10M, *optrA* was co-localized with *fexA*, *erm*(B), *tet*(M), and *tet*(L) on a novel 32,640 bp plasmid named pEfm10M-*optrA* (accession no. PZ148334) carrying replication genes of the Rep1 and Rep2 families. Comparative analysis revealed that pEfm10M-*optrA* exhibited the highest nucleotide homology (99.98% nucleotide identity; 61% coverage) to the uncharacterized plasmid 3 (accession no. LR134097.1) previously identified in *E. faecium* E1334 strain from the Netherlands (Fig. 4), including an identical *rep*_1_ gene. Notably, pEfm10M-*optrA* also showed partial similarity to the *E. faecalis* pRE25 (accession no. X92945) plasmid (49% coverage, 99.9% identity) and carried both its replication genes (*rep*_1_ and *rep*_2_), although the *rep*_1_ gene was interrupted by IS*1216*. Furthermore, similarly to pRE25, pEfm10M-*optrA* harbored the resistance genes *erm*(B), *tet*(M) and *tet*(L). Using the CONJscan tool, this plasmid was found to encode only a partial MobV family relaxase gene and was therefore likely non-self-transmissible (Table 2). The major ORFs' features are detailed in Table S2. In pEfm10M-*optrA* plasmid, *optrA* was co-localized with the *fexA* gene within a pseudo-compound transposon (PCTn) of 7,129 bp flanked by IS*1216* in the same orientation. This genetic organization has previously been described in pXM2013_42321 plasmid (accession no. MH225423) from *E. faecalis* XM2013_42321 strain (12).

**Fig 5:**
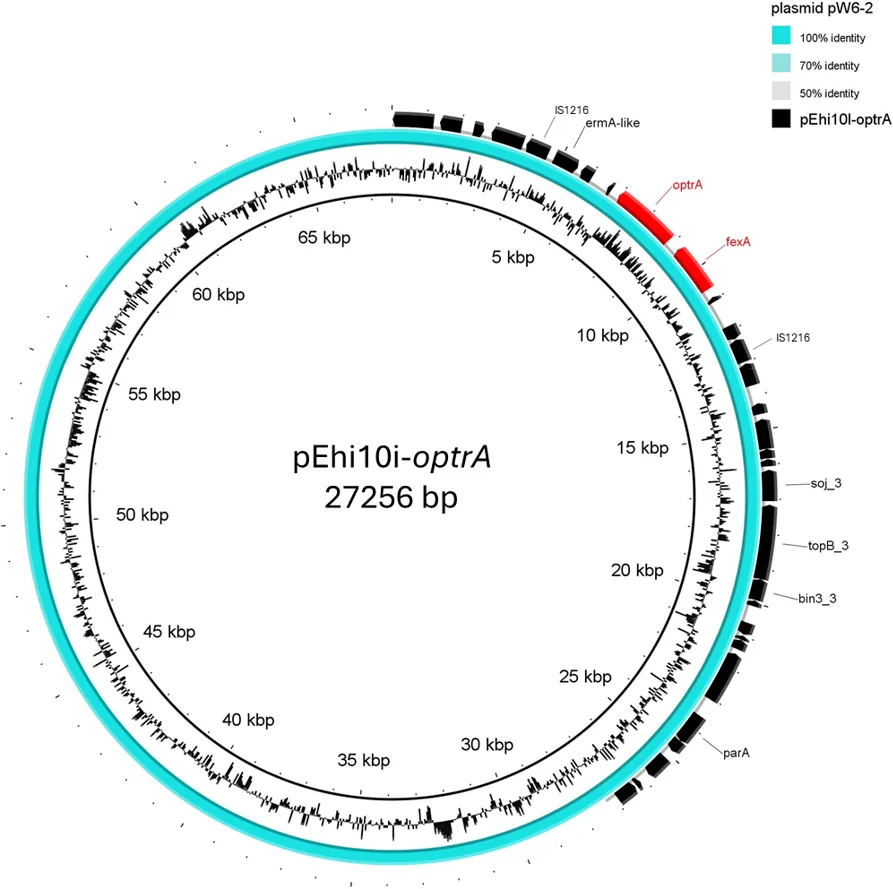
Schematic representation of pEhi10I-*optrA* plasmid (PZ148333) in *E. hirae* 10I. Plasmid was aligned with the similar pW6-2 plasmid (CP118549.1) detected in *E. faecium* W6 using BRIG software. Black arrows indicate the positions and orientations of genes; some antibiotic resistance determinants and relevant genes described in this study are shown in red.

In *E. hirae* 10I, *optrA* was co-localized with *fexA* and an *erm*(A)-like gene (a variant of *erm*(A) first described in *E. faecalis* by He et al. [33]) on a novel 27,256 bp plasmid belonging to the Inc18 incompatibility group, designated pEhi10I-*optrA* (accession no. PZ148333). This plasmid exhibited 99.97% nucleotide identity but 85% coverage with the larger pW6-2 plasmid (67,732 bp; accession no. CP118549.1) first detected in *E. faecium* W6 strain from sewage in China (Fig. 5). The pEhi10I-optrA plasmid is likely non-conjugative and non-mobilizable, as CONJscan analysis revealed the absence of genes associated with conjugation machinery (Table 2). The major ORF features are detailed in Table S3. As observed in *E. faecium* 10M, *optrA* was co-located with *fexA* and *erm*(A)-like within another PCTn of 9,190 bp flanked by IS*1216* in the same orientation. Similar structure has been previously described in p10-2-2 plasmid from *E. faecalis* 10-2-2 (accession no. KT862775) (12, 33).

### Transferability of oxazolidinone resistance genes

Conjugation experiments showed that three out of the four isolates successfully transferred the *optrA* gene in mating assays. In particular, *E. faecalis* 6AS, *E. faecium* 10M and *E. faecium* 8J were able to transfer *optrA* to the corresponding recipients, with transfer frequencies ranging from 2.03 × 10⁻⁶ to 6.36 × 10⁻⁴, in agreement with the finding that in all three strains *optrA* was located on conjugative or mobilizable plasmids and that all strains harbored an additional conjugative plasmid (Table 2). Moreover, in the mating experiment between *E. faecalis* 6AS and *E. faecalis* JH2-2, transconjugants acquired both linezolid resistance and high-level gentamicin resistance, as confirmed by MIC determination (Table 4). The highest frequency was observed in the intraspecies transfer from *E. faecium* 8J to *E. faecium* 64/3 (6.36 × 10⁻⁴) (Table 4).

**TABLE 4:** Phenotypes and genotypes of enterococcal donors and relevant transconjugants^*a*^

Donor	Oxazolidinone resistance gene	Recipient	Transfer frequency	Transconjugants					
				MIC (mg/L)					Oxazolidinone resistance gene
				FFC	CHL	LZD	TE	GEN	
*E. faecalis* 6AS	*optrA*	*E. faecalis* JH2-2	1.08 × 10^−5^	128	64	8	0.25	>512	*optrA*
*E. faecium* 8J	*optrA*	*E. faecium* 64/3	6.36 × 10^−4^	128	64	16	1	4	*optrA*
*E. faecium* 10M	*optrA*	*E. faecium* 64/3	2.03 × 10^−6^	128	64	16	1	4	*optrA*
*E. hirae* 10I	*optrA*	*E. faecium* 64/3	–^*b*^	–	–	–	–	–	–
*E. hirae* 10I	*optrA*	*E. faecalis* JH2-2	–	–	–	–	–	–	–

^*a*^ FFC, florfenicol; CHL, chloramphenicol; LZD, linezolid; TE, tetracycline; VA, vancomycin. *E. faecium* 64/3 MIC values: FFC (MIC, 4 mg/L), CHL (MIC, 4 mg/L), LZD (MIC, 1 mg/L), TE (MIC, 1 mg/L) and VA (MIC, 0.5 mg/L), GEN (MIC, 4 mg/L). *E. faecalis* JH2-2 MIC values: FFC (MIC, 1 mg/L), CHL (MIC, 4 mg/L), LZD (MIC, 1 mg/L), TE (MIC, 0.25 mg/L) and VA (MIC, 1 mg/L), GEN (MIC, 4 mg/L).

^*b*^ –, no data available due to unsuccessful conjugation.

Conversely, despite repeated attempts, *E. hirae* 10I was not able to transfer the *optrA* gene either to the *E. faecium* 64/3 recipient or to *E. faecalis* JH2-2, consistent with the lack of conjugative and mobilization functions in the pEhi10I-*optrA* plasmid.

### Detection of translocatable units and plasmid stability assays

Inverse PCR experiments revealed the presence of circular forms of the *optrA* genetic context only in the *E. faecium* 10M and *E. hirae* 10I isolates. Sanger sequencing of the amplicons revealed the presence of a single IS*1216* transposase in both PCTn elements.

Enterococcal isolates were serially passaged for 10 consecutive days on antibiotic-free BHIA to evaluate plasmid stability. Throughout this period, no changes in susceptibility to oxazolidinones and phenicols were observed, and no plasmid loss was detected by PCR assays.

### Phylogenetic analysis

**Fig 6:**
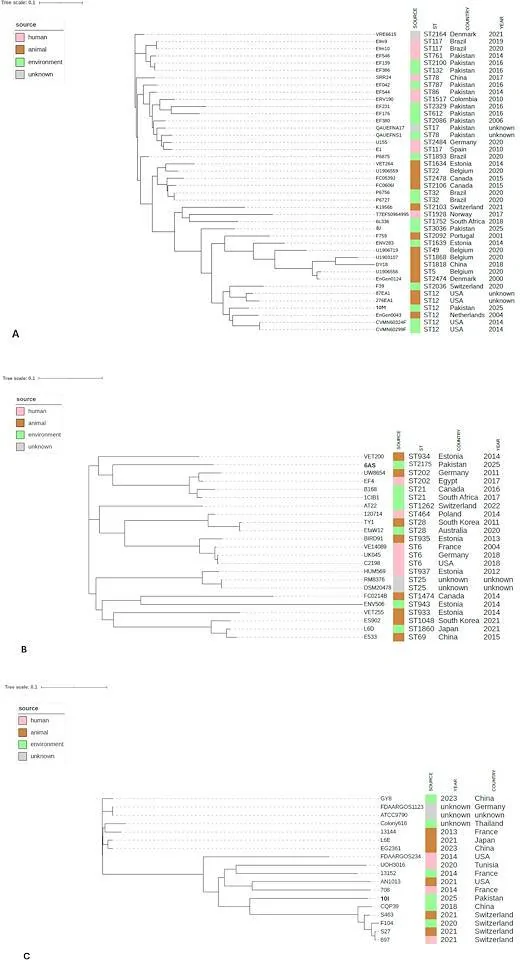
Phylogenetic trees containing 39 *Enterococcus faecium* (**A**), 22 *Enterococcus faecalis* (**B**), and 17 *Enterococcus hirae* (**C**) isolates from human, animal, and environmental sources, representing different countries and sequence types (STs). The tree was inferred by using the iTOL interactive user interface (https://itol.embl.de). The country of origin, the sequence type (ST), year of isolation, and source of each isolate are also shown. Scale bar indicates substitutions per site. The four strains investigated in the study are indicated in bold.

Comparison with *Enterococcus* genomes available in public repositories revealed that the Pakistani isolates primarily clustered according to their STs (Fig. 6).

Specifically, phylogenetic analysis of *E. faecium* genomes (Fig. 6A) revealed that *E. faecium* 10M and *E. faecium* 8J clustered together with strains of human, environmental, and animal origin within a genetically heterogeneous group comprising multiple STs. Despite this heterogeneity, ST12 isolates clustered together regardless of their geographic origin or source of isolation, confirming the evolutionary stability of this lineage. In particular, *E. faecium* 10M clustered within the ST12 clade together with five closely related genomes from the United States (87EA1, 276EA1, CVMN60324F, and CVMN60299F) and the Netherlands (EnGen0043), mainly of animal and environmental origin.

*E. faecium* 8J, belonging to the new ST3036, was closer to European strains, mainly of animal origin, previously identified in Portugal, Estonia, and Belgium, and belonging to different STs.

Interestingly, neither *E. faecium* 10M nor *E. faecium* 8J clustered with the other 10 *E. faecium* strains from Pakistan included in the analysis, which belonged to different STs and sources, indicating that geographic origin alone does not appear to be a major determinant of genomic relatedness among these *E. faecium* strains. An in-depth WGS analysis displayed that *E. faecium* 10M differs by 4,232 SNPs from *E. faecium* 8J, and both genomes exhibited 4,181–7,884 and 2,754–7,899 separating SNPs from the other *E. faecium* genomes, respectively.

Similarly, phylogenetic analysis of *E. faecalis* genomes revealed clustering primarily according to STs (Fig. 6B). Whole-genome SNP analysis showed that the *E. faecalis* 6AS genome exhibited 11,488–19,037 separating SNPs from the other *E. faecalis* genomes included in the data set. Notably, *E. faecalis* 6AS was most closely related to two enterococcal strains of animal origin, previously identified in Europe (UW8654 and Vet200; Estonia and Germany), two strains of environmental and human origin reported in Africa (1CIB1 and EF4; South Africa and Egypt), and an environmental isolate previously documented in Canada (B168) (Fig. 6B).

Finally, phylogenetic analysis of *E. hirae* genomes revealed a relatively diverse population structure, with isolates originating from different geographic regions and ecological sources, highlighting the wide ecological distribution of this species (Fig. 6C). Whole-genome SNP analysis showed that the genome of *E. hirae* 10I exhibited 10,838–13,120 separating SNPs from the other *E. hirae* genomes. Interestingly, it showed the closest phylogenetic relationship with four enterococcal strains (S463, F104, S27, and 697) previously reported in Switzerland, originating from animal, human, and environmental sources, as well as with the environmental isolate CQP39 identified in China, suggesting the presence of a small predominantly European cluster.

Similar to what was observed for *E. faecium*, the clustering pattern in *E. faecalis* and in *E. hirae* appeared to be independent of both the source of isolation and geographical origin.

## DISCUSSION

This study reports the recovery of *optrA*-positive enterococci from river sediments in the high-altitude Gilgit-Baltistan region of Pakistan, providing evidence that clinically relevant linezolid resistance genes can occur even in remote aquatic ecosystems with limited direct exposure to major urban or hospital effluents. These findings reinforce the concept that environmental dissemination of oxazolidinone resistance is not restricted to highly anthropized settings but may also involve geographically isolated environments potentially influenced by diffuse anthropogenic inputs and hydrological redistribution.

All selected isolates exhibited a multidrug-resistant phenotype and carried *optrA* on plasmids together with additional resistance determinants, most notably *fexA*, *erm* genes, and, in some cases, *tet* genes. This recurrent co-localization strongly suggests that co-selection may play a central role in the maintenance and spread of these plasmids in environmental enterococcal populations. From a One Health perspective, such multi-resistance platforms are particularly concerning as selective pressure exerted by one antimicrobial class can indirectly preserve resistance to critically important drugs used in human medicine.

The absence of 23S rRNA and L3/L4 mutations indicates that linezolid resistance in these isolates is mainly driven by transferable determinants rather than chromosomal target-site alterations. This is epidemiologically relevant because plasmid- and transposon-associated genes can spread horizontally across species and ecological compartments more readily than mutation-based resistance mechanisms. Consistent with previous reports (34), our findings support the growing importance of *optrA*, together with other acquired determinants, such as *poxtA* and *cfr*/*cfr*-like genes, in mediating oxazolidinone resistance in enterococci, often independently of classical 23S rRNA mutations. In environmental isolates, the mobilizable resistome may therefore represent a more relevant driver of oxazolidinone resistance than chromosomal adaptation. This is particularly noteworthy in Pakistan, where data on environmental oxazolidinone resistance remain extremely limited, but recent clinical genomic studies (27, 35) have already documented *optrA* among clinical enterococci, supporting the possibility of shared resistance pools between clinical and environmental sectors.

The marked genetic heterogeneity of the *optrA*-carrying plasmids identified in this study further supports the hypothesis that dissemination is driven by modular mobile genetic elements rather than by the expansion of a conserved resistance structure. Although *optrA* was plasmid-borne in all four isolates, it was embedded within distinct genetic platforms, including a truncated Tn*558*-related structure, a Tn*7363*-related region, and two IS*1216*-flanked pseudo-compound transposons. This diversity is highly consistent with previous reports showing that *optrA* is frequently associated with insertion sequences, especially IS*1216*, which appear to act as recombination hotspots promoting rearrangements, excision, and capture of resistance modules (12).

Consistently, the detection of mini-translocatable circular forms in two of the four isolates supports the active mobility of the *optrA* genetic context, as these intermediates are recognized hallmarks of recent or ongoing insertion sequence-mediated excision events, particularly in IS*1216*-associated regions, and suggest the potential for subsequent integration into new genetic locations. Although such circular intermediates were not detected in *E. faecalis* 6AS, the mobility of *optrA* genetic context in this strain may be related to its association with a mobilizable plasmid and to the presence of an additional conjugative plasmid. Overall, these findings indicate that river sediment enterococci can harbor genetically dynamic resistance platforms rather than merely reflecting residual contamination.

The conjugation results provide functional confirmation of the epidemiological relevance of these genetic findings. Three of the four isolates successfully transferred *optrA* to the recipient strain. Importantly, the failure of *E. hirae* 10I to transfer *optrA*, despite carrying an IS*1216*-associated platform, is likely attributable to the absence of conjugative or mobilizable elements required to mediate resistance transfer. This result underscores the importance of combining genomic predictions with functional assays when assessing AMR dissemination potential.

Equally important, all *optrA*-carrying plasmids remained stable after serial passages without antibiotic pressure, indicating that these resistance elements can persist in environmental bacterial communities even in the absence of direct selection. This stability likely reflects low fitness costs and/or the contribution of compensatory adaptation or plasmid maintenance systems, reinforcing the view that sediments act not only as transient sinks for resistant bacteria but also as long-term reservoirs where resistance determinants can persist and later disseminate under favorable conditions.

The phylogenetic analyses indicate that *optrA*-mediated oxazolidinone resistance is not confined to a single evolutionary lineage but occurs across genetically diverse *Enterococcus* backgrounds, supporting HGT rather than clonal expansion as the primary driver of dissemination across multiple ecological reservoirs. The four strains belonged to distinct species and STs (https://pubmlst.org/organisms). *E. faecium* ST12 is a rare ST mainly associated with poultry, food products, and occasionally environmental sources such as sewage (36). In addition, the identification of novel *E. faecalis* and *E. faecium* STs (ST2175 and ST3036, respectively) further expands the diversity of lineages known to harbor *optrA*. Notably, each isolate clustered with genomes of different geographic and ecological origins rather than forming a Pakistan-specific or site-specific clade, reinforcing the view that *optrA* is not restricted to a particular clonal lineage but can disseminate across diverse enterococcal genetic backgrounds. Altogether, these findings suggest that poorly characterized clones circulating in animal and environmental reservoirs may act as underrecognized carriers of transferable oxazolidinone resistance determinants and that surveillance focused primarily on well-known hospital-associated clones may underestimate the contribution of “silent” lineages to the broader environmental and global resistome. Taken together, this phylogenetic heterogeneity, coupled with the absence of a site-specific clustering pattern, suggests that local environmental dissemination is more likely driven by multiple introductions and ecological redistribution processes than by the persistence of a single endemic clone.

In a mountainous region, such as Gilgit-Baltistan, hydrological connectivity, seasonal runoff, tourism-related pressure, livestock activity, and localized wastewater inputs may all contribute to the introduction and redistribution of resistant bacteria, even when direct industrial or hospital inputs are limited. Although these sources were not directly assessed in the present study, the detection of mobile and stable *optrA*-carrying plasmids in sediment enterococci strongly supports the need to further explore the environmental pathways sustaining their persistence and dissemination.

In conclusion, the present findings underscore that remote river sediments can harbor clinically relevant oxazolidinone resistance determinants embedded in mobile multidrug-resistance platforms and that such environments may serve as underrecognized reservoirs and dissemination nodes for linezolid resistance. These results reinforce the importance of incorporating remote aquatic ecosystems into One Health AMR surveillance frameworks, particularly in regions where environmental pathways remain poorly characterized but may facilitate the silent circulation of clinically relevant resistance genes.

## MATERIALS AND METHODS

### Sampling sites, sample processing, and bacterial isolation

Sampling was conducted in July 2024 in the Skardu District of Gilgit-Baltistan, Pakistan. Sampling sites were selected across three main areas: the surroundings of Skardu city, the Shigar River Valley, and the Braldo River Valley, with Askole representing the uppermost region of the study area. Sediment samples were collected from natural and artificial basins, primary and secondary watercourses, and springs across these regions. A total of 12 samples were collected (Fig. 6). All samples were incubated overnight at 37°C in azide broth (Oxoid, Basingstoke, UK) for the selective enrichment of enterococci.

An aliquot (100 μL) of each enrichment culture was spread on Slanetz Bartley agar plates supplemented with florfenicol (10 mg/L) for the selection of resistant enterococcal isolates. From each selective agar plate, eight presumptive florfenicol-resistant enterococcal colonies were randomly picked for further analysis.

### Genotypic and phenotypic characterization

Selected florfenicol-resistant enterococci were screened by PCR assays for the presence of *cfr*, *optrA*, and *poxtA* genes using primer pairs previously described (37).

Isolates carrying linezolid resistance genes were identified by MALDI-TOF (Vitek-MS, bioMérieux, Marcy l'Étoile, France) and tested for their susceptibility to florfenicol, chloramphenicol, linezolid, vancomycin, erythromycin, tetracycline (Sigma Aldrich, St. Louis, MI, USA) by broth microdilution assay following the standard EUCAST methodology, and susceptibility results were interpreted according to the EUCAST clinical breakpoints (version 15.0, 2025; http://www.eucast.org). Since no EUCAST breakpoint is currently available for florfenicol, an MIC value of 16 mg/L was adopted as the resistance breakpoint. *E. faecalis* ATCC 29212 was used as quality control.

### WGS and sequence analysis

Bacterial genomic DNA was extracted by the QIAcube automated extractor using DNeasy PowerLyzer PowerSoil Kit according to the manufacturer’s instructions (Qiagen, Germany). Extracted DNA was subjected to WGS using both the short-read Illumina MiSeq platform (Illumina Inc., San Diego, CA, USA) with a 2 × 150 bp paired-end and a long-read MinION, Oxford Nanopore Technologies sequencing approach (MicrobesNG, Birmingham, UK). Unicycler v.0.4.8 software was used for the hybrid assembly of short and long reads (https://github.com/rrwick/Unicycler). The RAST tool (https://rast.nmpdr.org/) was used for the annotation of DNA sequences.

*In silico* identification of acquired antimicrobial resistance genes, molecular typing, and phylogenetic analysis were carried out using dedicated tools (PlasmidFinder 2.1, ResFinder v.3.2, LRE-Finder 1.0, MLST 2.0, and VirulenceFinder 2.0) available at the Center for Genomic Epidemiology (available at https://www.genomicepidemiology.org/) using default parameters, and by the BLAST site (https:// blast.ncbi.nlm.nih.gov/Blast.cgi). Average nucleotide identity (ANI) analysis was performed using the FastANI calculator tool (available at https://gtdb.ecogenomic.org/tools/fastani). CONJscan tool (https://galaxy.pasteur.fr/) was used to screen all plasmids for genes encoding key components of conjugation systems.

### Conjugation experiments

Conjugal transfer was performed on membrane filters as previously described (38). In mating experiments, all isolates carrying linezolid resistance genes were used as donors, while *E. faecium* 64/3 and *E. faecalis* JH2-2 were used as recipients. Transconjugants were selected on brain heart infusion agar (Oxoid) containing florfenicol (10 mg/L), fusidic acid (25 mg/L), and rifampicin (25 mg/L). The resulting colonies were tested for the presence of linezolid resistance genes by PCR and for susceptibility to florfenicol and linezolid. SmaI-PFGE was performed as previously described (39), and banding patterns were analyzed to confirm the genetic background of the transconjugants. Conjugation frequencies were expressed as the number of transconjugants per recipient cell.

### Detection of mini translocatable units

To investigate the excision of genetic contexts carrying the *optrA* gene, PCR assays were performed using outward-facing primer pairs (optrAdiv-FW 5′- GAAAAATAACACAGTAAAAGGC-3′ and optrAdiv-RV 5′-TTTTTCCACATCCATTTCTACC-3′) targeting the *optrA* gene. The *optrA*-carrying *E. faecium* E35048 (40) isolates was used as positive control.

### Plasmid stability

Plasmid stability over time (10 days) was evaluated by performing daily serial passages of isolates carrying linezolid resistance genes on antibiotic-free BHIA, at 37°C. After each overnight passage, 10 colonies were randomly chosen and tested for their susceptibility to oxazolidinones and phenicols. Genomic DNA was then extracted from each isolate and screened by PCR for the presence of linezolid resistance genes.

### Phylogenetic analysis

A comparison with publicly available genome sequences was performed in order to investigate if the strains of this study showed similarities with environmental or clinical strains, as well as to identify possible geographical correlations. Seventy-eight complete genomes available in the NCBI database were downloaded, including *E. faecium* (*N* = 39), *E. faecalis* (*N* = 22), and *E. hirae* (*N* = 17). The final data set comprised enterococcal genomes from human, animal, and environmental sources, representing different countries and STs. Phylogenetic relatedness of the sequenced isolates was evaluated using CSI Phylogeny 1.4 (https://cge.cbs.dtu.dk/services/CSIPhylogeny/), with default parameters, to infer phylogenetic trees from assembled WGS data. The *E. faecium* SRR24 (GenBank accession no. CP038996.1), *E. faecalis* T5 (GenBank accession no. GCA_000393015.1), and *E. hirae* FDAARGOS_1123 (GenBank accession no. CP068483.1) genomes were used as reference genomes.

Genomes were compared with the isolates obtained in this study by constructing a phylogenetic tree based on the concatenated alignment of the SNPs. The tree was manually annotated using iTOL (v.6, https://itol.embl.de/; access date: 10 February 2026).

## Data Availability

The WGS data of *E. faecium* 8J and 10M and *E. faecalis* 6AS and *E. hirae* 10I are available under BioProject ID PRJNA1392913, and the nucleotide sequences of the plasmids pEfa6AS-*optrA*, pEfm10M-*optrA,* and pEhi10I-*optrA* have been deposited in GenBank under accession numbers PZ148336, PZ148334, and PZ148333, respectively.

## SUPPLEMENTAL MATERIAL

Supplemental material aem.01129-26-s0001.docx Tables S1 to S3.

ASM does not own the copyrights to Supplemental Material that may be linked to, or accessed through, an article. The authors have granted ASM a non-exclusive, world-wide license to publish the Supplemental Material files. Please contact the corresponding author directly for reuse.
