# Supplementary material for: Transferable optrA-mediated linezolid resistance in enterococci from high-altitude river sediments of Gilgit-Baltistan, Pakistan
Source: Appl Environ Microbiol. 2026 Sep 2;92(9):e01129-26. doi: 10.1128/aem.01129-26 (PMC13599501; doi:10.1128/aem.01129-26)
Supplement: Supplementary file 1 [file aem.01129-26-s0001.docx]

**Table S1.** Amino acid sequence identities/similarities of putative proteins encoded by pEfa6AS-*optrA* plasmid (GenBank accession no. PZ148336).

----------------------------------------------------------------------------------------------------------------------------------------------------------------------------------------------------------------------------------------------------	BLASTP analysis*a*	Size	--------------------------------------------------------------------------------------------------------------------------------------------

ORF	Start	Stop	(amino	Predicted function	% Amino acid

(bp)	(bp)	acids)	Most significant database match	Accession no.	identity (% amino

acid similarity)

----------------------------------------------------------------------------------------------------------------------------------------------------------------------------------------------------------------------------------------------------

*orf1*	1	1014	337	Replication initiator protein A	replication initiator protein A [*Enterococcus*]	WP_002397973.1	100 (100)

*orf2*	1145	1813	222	CPBP family intramembrane glutamic	type II CAAX endopeptidase family protein [*E. faecalis*]	MDT2096075.1	100 (100)	endopeptidase

*orf3*	1856	2908	350	Peptide ABC transporter substrate-binding	peptide ABC transporter substrate-binding protein [*E. faecalis*]	ELU9001701.1	99 (99)

protein

*orf4*	2963	3544	193	IS3 family transposase ISLmo5	IS3 family transposase, partial [*E. faecalis*]	EOJ08291.1	100 (100)

*orf5*	3796	4089	97	hypothetical protein [*E. faecalis*]	EOJ08292.1	100 (100)

*orf6*	4228	4836	202	Septal ring factor EnvC, activator of murein	hypothetical protein [*E. faecalis*]	WP_064686955.1	100 (100)

hydrolases AmiA and AmiB

*orf7*	4995	5480	161	PcfB family protein [*E. faecalis*]	WP_002386493.1	100 (100)

*orf8*	5480	7309	609	Type IV secretion/DNA transfer system	VirD4-like conjugal transfer protein, CD1115 family [*E. faecalis*]	WP_325826353.1	99 (100)

ATPase VirD4/TraG/TraD

*orf9*	7360	8538	392	toprim domain-containing protein	ArdC-like ssDNA-binding domain-containing protein [*E. faecalis*]	HIM4106395.1	99 (99)

*orf10*	8539	9497	320	Antirestriction protein ArdC	ArdC-like ssDNA-binding domain-containing protein [*E. faecalis*]	WP_107164625.1	99 (100)

*orf11*	9530	9802	90	hypothetical protein [*E. faecalis*]	EOE6364433.1	100 (100)

*orf12*	10048	10404	118	plasmid mobilization relaxosome protein MobC [*Enterococcus*]	WP_010784124.1	100 (100)

*orf13*	10405	12090	561	relaxase/mobilization nuclease	relaxase/mobilization nuclease domain-containing protein [*Enterococcus*]	WP_010784125.1	99 (99)

domain-containing protein

*orf14*	12123	12473	116	hypothetical protein [*E. faecalis*]	WP_010784126.1	100 (100)

*orf15*	13161	14354	397	PcfJ domain-containing protein [*E. faecalis*]	NSQ25770.1	100 (100)

*orf16*	14354	15127	257	Cas9 inhibitor AcrIIA9 family protein [*Enterococcus*]	WP_002362401.1	100 (100)

*orf17*	15232	15822	196	hypothetical protein [*Enterococcus*]	WP_002362400.1	100 (100)

*orf18*	15846	16019	57	DpnD/PcfM family protein *[E. faecalis*]	EEU24425.1	100 (100)

*orf19*	16022	16207	61	hypothetical protein [*E. faecalis*]	EEI56238.1	98 (100)

*orf20*	16805	17035	76	hypothetical protein [*E. faecalis*]	WP_002360849.1	100 (100)

*orf21*	17249	19363	704	putative ATP-dependent Clp protease	ATP-dependent protease ClpL [*Enterococcus*]	WP_002362396.1	100 (100)

ATP-binding subunit

*orf22*	20061	20315	84	transposase, partial [*E. faecium*]	WP_002301418.1	100 (100)

*orf23*	20366	21187	186	IS3 family transposase ISBce13	recombinase family protein [*E. faecium*]	MBE8866018.1	99 (100)

*orf24*	21240	21800	186	Putative transposon Tn552 DNA-invertase	recombinase family protein [*E. faecium*]	MBE8866018.1	99 (100)

bin3

*orf25*	21797	23227	476	hypothetical protein [*E. faecalis*]	MBK4753805.1	100 (100)

*orf26*	23389	24084	231	IS256 family transposase ISEfm2	IS256-like element ISEfm2 family transposase [*E. faecium*]	HBM6029058.1	100 (100)

*orf27*	24327	24686	119	IS30 family transposase IS658	hypothetical protein [*E. faecalis*]	EOJ78277.1	100 (100)

*orf28*	24899	25168	89	helix-turn-helix domain-containing protein [*Enterococcus*]	WP_010784111.1	100 (100)

*orf29*	25784	27646	620	Superfamily II DNA or RNA helicase,	DEAD/DEAH box helicase [*E. faecalis*]	WP_270785781.1	99 (100)

SNF2 family

*orf30*	27643	28860	405	hypothetical protein [*E. faecalis*]	WP_002403427.1	99 (100)

*orf31*	28865	31459	864	DNA-directed RNA polymerase, sigma	sigma factor-like helix-turn-helix DNA-binding protein [Bacilli]	WP_002362439.1	100 (100)

subunit (sigma70/sigma32)

*orf32*	31864	33147	427	hypothetical protein [*E. faecalis*]	WP_002405123.1	99 (100)

*orf33*	33253	34104	283	IS3 family transposase ISBce13	IS3 family transposase [*E. faecalis*]	WP_010784113.1	100 (100)

*orf34*	34155	34409	84	transposase [*E. faecalis*]	WP_025188193.1	96 (97)

*orf35*	34548	35150	200	Protein adenylyltransferase NmFic	protein adenylyltransferase Fic, partial [*E. faecalis*]	WP_033600231.1	99 (100)

*orf36*	35415	36353	312	hypothetical protein [*E. faecalis*]	WP_425136880.1	97 (97)

*orf37*	36465	36806	113	type II toxin-antitoxin system PemK/MazF family toxin [*E. faecalis*]	WP_010715691.1	99 (100)

*orf38*	36807	37036	76	type II toxin-antitoxin system PemI/MazE family antitoxin [*Enterococcus*]	WP_010784115.1	100 (100)

*orf39*	37264	37860	198	Recombinase family protein	recombinase family protein [*E. faecalis*]	WP_408649489.1	98 (99)

*orf40*	37877	38161	94	hypothetical protein [*E. faecalis*]	WP_002394798.1	99 (100)

*orf41*	38163	38396	77	hypothetical protein [*E. faecalis*]	WP_227337614.1	99 (100)

*orf42*	38556	38810	84	hypothetical protein [*E. faecalis*]	WP_010818063.1	94 (97)

*orf43*	38927	39595	222	CPBP family intramembrane glutamic	CPBP family intramembrane glutamic endopeptidase [*Enterococcus*]	WP_002397511.1	99 (99)

endopeptidase

*orf44*	39631	39948	105	UPF0145 protein BCE33L0904	heavy metal-binding domain-containing protein [*E. faecalis*]	WP_002394802.1	99 (99)

*orf45*	40551	43523	990	Tn3 family transposase TnBth1	Tn3 family transposase [*E. faecalis*]	WP_104681140.1	99 (99)

*orf46*	43654	44259	201	Putative transposon Tn552 DNA-invertase	recombinase family protein [*E. faecalis*]	WP_161994384.1	99 (99)

bin3

*orf47*	44648	45613	321	reverse transcriptase family protein	reverse transcriptase family protein [*Enterococcus*]	WP_065813860.1	100 (100)

*orf48*	45594	45875	93	hypothetical protein [*Enterococcus*]	WP_065813861.1	100 (100)

*orf49*	46383	46961	192	Serine recombinase PinR	recombinase family protein [*Enterococcus*]	WP_065813862.1	100 (100)

*orf50*	46995	47630	211	hypothetical protein [*E. faecalis*]	AKA86781.1	100 (100)

*orf51*	47740	48135	131	DUF5388 domain-containing protein [*Enterococcaceae*]	WP_065813864.1	100 (100)

*orf52*	48143	48973	276	ParA family protein	ParA family protein [*E. faecium*]	WP_231432272.1	99 (99)

*orf53*	50208	50936	242	Adenine-specific DNA methylase	DNA adenine methylase, partial [*E. faecium*]	WP_065813866.1	100 (100)

*orf54*	51882	53795	637	ABC-F type ribosomal protection protein	ABC-F type ribosomal protection protein OptrA [*E. faecalis*]	WP_342834678.1	100 (100)

OptrA

*orf55*	54168	54359	63	hypothetical protein [*E. faecalis*]	HIB1849985.1	100 (100)

*orf56*	54537	55964	475	chloramphenicol/florfenicol efflux MFS	chloramphenicol/florfenicol efflux MFS transporter FexA [*E. faecalis*]	WP_319464845.1	100 (100)

transporter FexA

*orf57*	57068	57505	145	hypothetical protein [*E. faecalis*]	WP_349788343.1	99 (100)

*orf58*	57746	58117	123	hypothetical protein [*Enterococcus*]	WP_366533289.1	100 (100)

*orf59*	58141	59436	431	DNA polymerase IV	Y-family DNA polymerase [Bacilli]	WP_105461294.1	99 (100)

*orf60*	59440	62358	972	Tn3 family transposase Tn4430	Tn3 family transposase [*E. cecorum*]	WP_047341018.1	99 (100)

*orf61*	62362	62916	184	Recombinase family protein	recombinase family protein [*E. faecalis*]	WP_187417939.1	99 (99)

*orf62*	63271	64008	245	rRNA adenine N-6-methyltransferase	23S rRNA (adenine(2058)-N(6))-methyltransferase Erm(B) [*E. faecium*]	WP_088786634.1	99 (99)

*orf63*	64680	65135	151	hypothetical protein [*E. faecalis*]	WP_248878851.1	94 (98)

*orf64*	65705	66001	98	type III secretion system protein PrgN [*E. faecalis*]	EJV08693.1	98 (98)

*orf65*	66239	67021	260	ParA family protein	ParA family protein [*E. faecalis*]	WP_279256097.1	99 (99)

*orf66*	67014	67370	118	hypothetical protein [*E. faecalis*]	WP_182924528.1	97 (99)

--------------------------------------------------------------------------------------------------------------------------------------------------------------------------------------------------------------------------------------------

*^a^*For each ORF, only the most significant identity detected is listed

**Table S2.** Amino acid sequence identities/similarities of putative proteins encoded by pEfm10M-*optrA* plasmid (GenBank accession no. PZ148334).

----------------------------------------------------------------------------------------------------------------------------------------------------------------------------------------------------------------------------------------------------	BLASTP analysis*a*	Size	--------------------------------------------------------------------------------------------------------------------------------------------

ORF	Start	Stop	(amino	Predicted function	% Amino acid

(bp)	(bp)	acids)	Most significant database match	Accession no.	identity (% amino

acid similarity)

----------------------------------------------------------------------------------------------------------------------------------------------------------------------------------------------------------------------------------------------------

*orf1*	1432	2580	382	IS200/IS605 family transposase ISEfa4	IS200/IS605 family element RNA-guided endonuclease TnpB	WP_069722873.1	99 (99)

[*Enterococcus faecium*]

*orf2*	2597	3001	134	IS200/IS605-like element ISEfa4 family transposase [*E. faecium*]	WP_060799090.1	99 (99)

*orf3*	3087	4283	398	MobM_relaxase	MobV family relaxase, partial [*E. faecalis*]	WP_010782938.1	100 (100)

*orf4*	4847	6223	458	tetracycline efflux MFS transporter Tet(L)	tetracycline efflux MFS transporter Tet(L) [*Staphylococcus aureus*]	WP_012779616.1	99 (99)

*orf5*	6417	8336	639	tetracycline resistance ribosomal protection	tetracycline resistance ribosomal protection protein Tet(M) [*E. faecium*]	WP_317728076.1	98 (99)

protein Tet(M)

*orf6*	8713	9330	205	conjugative transposon protein TcpC	conjugal transfer protein, partial [*E. faecium*]	WP_311798573.1	100 (100)

*orf7*	9507	10187	226	IS6 family transposase IS1216E	IS6-like element IS1216 family transposase [*E. hirae*]	WP_002354485.1	100 (100)

*orf8*	10456	12369	637	ABC-F type ribosomal protection protein	ABC-F type ribosomal protection protein OptrA [*E. faecalis*]	WP_342834678.1	100 (100)

OptrA

*orf9*	12742	12933	63	hypothetical protein [*E. faecalis*]	HIB1849985.1	99 (100)

*orf10*	13111	14538	475	chloramphenicol/florfenicol efflux MFS	chloramphenicol/florfenicol efflux MFS transporter FexA [*E. faecalis*]	WP_319464845.1	100 (100)

transporter FexA

*orf11*	15827	16507	226	IS6 family transposase IS1216E	transposase [*E. faecalis*]	OTP41372.1	99 (100)

*orf12*	16549	17235	228	primase C-terminal domain-containing protein, partial [*E. faecium*]	WP_252706267.1	99 (99)

*orf13*	17247	17687	146	DNA topoisomerase 1	type IA DNA topoisomerase, partial [*E. faecalis*]	WP_023042683.1	100 (100)

*orf14*	17827	18090	87	peptide-binding protein [*E. faecium*]	WP_425211801.1	100 (100)

*orf15*	18319	19056	245	rRNA adenine N-6-methyltransferase	23S rRNA (adenine(2058)-N(6))-methyltransferase Erm(B) [*S. suis*]	WP_269088772.1	99 (100)

*orf16*	19327	19650	107	DUF5388 domain-containing protein [*E. faecium*]	WP_113852518.1	99 (99)

*orf17*	19643	20146	167	ParA family protein, partial [*E. lactis*]	WP_269400482.1	99 (100)

*orf18*	20272	20952	226	IS6 family transposase IS1216E	transposase [*E. faecalis*]	OTP41372.1	99 (100)

*orf19*	21150	21755	201	Protein adenylyltransferase NmFic	protein adenylyltransferase Fic [*E. gallinarum*]	WP_257603586.1	94 (97)

*orf20*	21771	22343	190	Serine recombinase PinR	recombinase family protein [*Enterococcus*]	WP_135172343.1	96 (98)

*orf21*	22816	23238	140	IS3 family transposase ISBce13	DDE-type integrase/transposase/recombinase, partial [*S. aureus*]	WP_248499421.1	99 (100)

*orf22*	23489	24325	278	IS3 family transposase IS1485	IS3 family transposase [*E. faecalis*]	WP_108119894.1	100 (100) *orf23*	24361	24651	96	IS3 family transposase IS1485	transposase [*E. faecium*]	ELA78001.1	99 (98)

*orf24*	25057	25311	84	transposase, partial [*E. faecium*]	WP_234698068.1	100 (100)

*orf25*	25504	25779	91	DNA segregation protein PrgO [*E. faecium*]	WP_408588678.1	100 (100)

*orf26*	25751	26704	317	ParA DNA segregation protein	ParA family protein [*E. faecium*]	WP_086324098.1	98 (99)

*orf27*	27316	28809	497	primase C-terminal domain-containing protein	primase C-terminal domain-containing protein [*S. aureus*]	WP_242414046.1	99 (99)

*orf28*	28944	29240	98	type III secretion system protein PrgN [*E. durans*]	WP_311931566.1	99 (100)

*orf29*	29264	30223	319	IS30 family transposase IS1062	IS30 family transposase, partial [*E. faecium*]	WP_318760692.1	100 (100)

*orf30*	30406	31257	283	hypothetical protein [*E. faecalis*]	WP_048962559.1	99 (98)

*orf31*	31278	31490	70	hypothetical protein [*E. faecium*]	WP_080475310.1	100 (100)

*orf32*	31923	32603	226	IS6 family transposase IS1216E	transposase [*E. faecalis*]	OTP41372.1	99 (100)

----------------------------------------------------------------------------------------------------------------------------------------------------------------------------------------------------------------------------------------------

*^a^*For each ORF, only the most significant identity detected is listed

**Table S3.** Amino acid sequence identities/similarities of putative proteins encoded pEhi10I-*optrA* plasmid (GenBank accession no. PZ148333).

----------------------------------------------------------------------------------------------------------------------------------------------------------------------------------------------------------------------------------------------------	BLASTP analysis*a*	Size	--------------------------------------------------------------------------------------------------------------------------------------------

ORF	Start	Stop	(amino	Predicted function	% Amino acid

(bp)	(bp)	acids)	Most significant database match	Accession no.	identity (% amino

acid similarity)

----------------------------------------------------------------------------------------------------------------------------------------------------------------------------------------------------------------------------------------------------

*orf1*	18	1196	392	IS256 family transposase ISEf1	IS256-like element ISEf1 family transposase [*Enterococcus faecium*]	WP_227454415.1	99 (98)

*orf2*	1373	2005	210	pentapeptide repeat-containing protein [*E. faecium*]	WP_104870534.1	99 (100)

*orf3*	2356	2655	99	bacteriocin immunity family protein [*E. faecium*]	WP_070828003.1	100 (100)

*orf4*	2878	3834	318	IS256 family transposase ISEf1	IS256-like element ISEf1 family transposase [*E. faecium*]	WP_142972427.1	99 (100)

*orf5*	3926	4606	226	IS6 family transposase IS1216E	IS6-like element IS1216 family transposase [*Lactobacillales*]	WP_060797357.1	99 (99)

*orf6*	4808	5539	243	rRNA adenine N-6-methyltransferase	23S rRNA (adenine(2058)-N(6))-methyltransferase Erm(A)	WP_381387613.1	100 (100)

[*Staphylococcus aureus*]

*orf7*	5664	6026	120	EFR1 family ferrodoxin [*Campylobacter coli*]	WP_264378400.1	99 (100)

*orf8*	6956	8869	637	ABC-F type ribosomal protection protein	ABC-F type ribosomal protection protein OptrA [*E. faecalis*]	WP_342834678.1	99 (99)

OptrA

*orf9*	9239	10666	475	chloramphenicol/florfenicol efflux MFS	chloramphenicol/florfenicol efflux MFS transporter FexA [*E. faecalis*]	WP_319464845.1	100 (100)

transporter FexA

*orf10*	11868	12284	138	N-6 DNA methylase, partial [*E. faecium*]	WP_311808164.1	100 (100)

*orf11*	12307	12987	226	IS6 family transposase IS1216E	IS6-like element IS1216 family transposase [*Lactobacillales*]	WP_060797357.1	99 (99)

*orf12*	13041	13256	71	DpnD/PcfM family protein, partial [*E. gallinarum*]	WP_349701227.1	100 (100)

*orf13*	13198	13695	165	DnaJ domain-containing protein [*E. hailinensis*]	WP_407857822.1	97 (98)

*orf14*	14229	14546	105	hypothetical protein [*S. aureus*]	WP_445977830.1	100 (100)

*orf15*	14693	15556	287	Toxin zeta	zeta toxin family protein [*Enterococcaceae*]	WP_125276263.1	97 (99)

*orf16*	15558	15830	90	Antitoxin epsilon	antitoxin [*Enterococcus*]	WP_250699067.1	100 (100)

*orf17*	15847	16056	69	Omega Transcriptional Repressor	peptide-binding protein [*E. faecium*]	WP_251843985.1	97 (98)

*orf18*	16154	17050	298	ParA family protein	ParA family protein [*S. suis*]	WP_318821906.1	96 (97)

*orf19*	17153	19297	714	DNA topoisomerase 3	type IA DNA topoisomerase [*Enterococcus*]	WP_231477931.1	97 (97)

*orf20*	19297	19914	205	Putative transposon Tn552 DNA-invertase	recombinase family protein [*E. thailandicus*]	WP_270345033.1	97 (98)

bin3

*orf21*	19928	20098	56	hypothetical protein [*Lactobacillales*]	WP_172599325.1	98 (100)

*orf22*	20600	20956	118	IS6 family transposase IS1216V	IS6-like element IS1216 family transposase, partial [*E. faecium*]	WP_113847467.1	98 (100)

*orf23*	21122	21409	95	type III secretion system protein PrgN [*E. faecalis*]	WP_241408351.1	97 (98)

*orf24*	21545	23044	499	Primase C terminal 1	primase C-terminal domain-containing protein [*Enterococcus*]	WP_412247230.1	99 (99)

*orf25*	23595	24506	303	Chromosome partitioning protein ParA	ParA family protein [*E. faecium*]	WP_194179438.1	99 (100)

*orf26*	24508	24879	123	hypothetical protein [*E. faecium*]	WP_142967633.1	100 (100)

*orf27*	25142	25822	226	IS6 family transposase IS1216E	IS6-like element IS1216 family transposase [*Lactobacillales*]	WP_060797357.1	99 (99)

*orf28*	26135	26287	50	hypothetical protein [*Enterococcus*]	WP_002364543.1	100 (100)

*orf29*	26336	26935	199	Tyrosine recombinase XerC	tyrosine-type recombinase/integrase [*E. italicus*]	WP_007209378.1	98 (98)

----------------------------------------------------------------------------------------------------------------------------------------------------------------------------------------------------------------------------------------------

*^a^*For each ORF, only the most significant identity detected is listed
